# The correlation and role analysis of KCNK2/4/5/15 in Human Papillary Thyroid Carcinoma microenvironment

**DOI:** 10.7150/jca.45604

**Published:** 2020-06-29

**Authors:** Xu Lin, Jing-Fang Wu, Dong-Mei Wang, Jing Zhang, Wen-Jing Zhang, Gang Xue

**Affiliations:** 1 Department of Histology and Embryology, Hebei North University, Zhangjiakou, 075000, China.; 2 Department of Otorhinolaryngology Head and Neck Surgery, Hebei North University, Zhangjiakou, 075000, China.

**Keywords:** papillary thyroid carcinoma, KCKNs, microenvironment, immune cell infiltration

## Abstract

**Background:** KCNKs, potassium two pore domain channel family K members, can maintain the resting potential, regulate the amplitude and duration of the plateau of the action potential, and change the membrane potential and membrane excitability. Evidence from many studies indicates that KCNKs is abnormally expressed in many solid tumors and plays a regulatory role in the development and malignant progression of cancer. However, the expression pattern and prognostic value of KCNK factors in papillary thyroid carcinoma have not been reported.

**Methods:** In this study, we used the data from databases such as ONCOMINE, GEPIA, Kaplan-Meier Plotter, and cBioPortal to perform bioinformatics analysis of KCNK factors in patients with thyroid cancer.

**Results:** We found that the mRNA expression of KCNK1, KCNK5, KCNK6, KCNK7, and KCNK15 were significantly higher in thyroid cancer tissues than that in normal tissues, while KCNK2, KCNK4, KCNK9, KCNK16 and KCNK17 mRNA levels were decreased compared to normal tissues. And the expression levels of KCNK1/2/4/5/6/7/15 were correlated with the tumor stage. Survival analysis using the Kaplan-Meier Plotter database revealed that KCNK2/3/4/5/12/15 were associated with overall survival (OS) in patients with thyroid cancer.

**Conclusion:** Finally, the results of ROC curves, immunohistochemical staining, immune cell infiltration and kinase / miRNA / transcription factor regulation showed that KCNK2, KCNK4, KCNK5 and KCNK15 levels could be used as biomarkers for PTC diagnosis. This study implied that KCNK2, KCNK4, KCNK5 and KCNK15 are potential targets of precision therapy for patients with thyroid cancer and these genes are new biomarkers for the therapeutic target for thyroid cancer.

## Introduction

Due to the continuous improvement of modern diagnostic methods and other factors, thyroid cancer (TC) has become one of the common malignant tumors 1. It is estimated that there are 53,990 new cases of thyroid cancer in the US in 2018, and thyroid cancer was the fifth most common malignant tumor in American women 2. In China's 2018 cancer report, it was mentioned that thyroid cancer ranked fourth in female tumors in 2014. Among them, papillary thyroid cancer (PTC) is the most common pathological type, accounting for about 80% of the total number of thyroid cancers 3. Although thyroid cancer, including PTC, has the advantages of high diagnostic detection rate, good therapeutic effect, and high survival rate 4. However, once distant metastasis of PTC occurs, it will seriously affect the survival time of patients, and some researchers have reported that the five year survival rate drops to about 54.1% for patients with thyroid cancer due to distant metastasis 5-7. Therefore, early detection of lymph node metastases and the study of the molecular mechanism of thyroid cancer lymph node metastasis are of great significance for the choice of surgical approach, clinical treatment and prognosis of thyroid cancer patients.

The relationship between ion channels and ion channels has become a new focus of attention with the development of thyroid cancer research. A large amount of evidence indicates that the generation, malignant transformation and metastasis of many solid tumors can be regulated by ion channels. The KCNK channel was originally called the K2P channel, and was later named the KCNK channel. Unlike voltage-dependent potassium channels and inwardly rectifier potassium channels, KCNK channels have four transmembrane segments (TM) and two membrane domains (P) 8. There are currently 15 subfamilies of the KCNK family, which are divided into 5 groups (TASK, THIK, TWIK, TREK, TALK channels) according to their sequence homology and functional similarity 9. KCNKs have the effect of maintaining resting potentials, affecting the amplitude and duration of the plateau phase of action potentials, and transmitting various extracellular stimuli into cells, thereby causing changes in membrane potential and membrane excitability 9, 10. Based on the above-mentioned effects, KNCKs can participate in the regulation of cell cycle, protein transport, metabolic regulation, and other cellular processes under physiological conditions 11, 12. Studies have pointed out that ion channels play an important role in driving the malignant transformation of tumor cells, especially in promoting the malignant proliferation of cancer cells. For example, double-pore domain potassium channel KCNK9 is highly expressed in breast and lung cancer 13. Similarly, KCNK2 is more highly expressed in prostate cancer tissues than in adjacent tissues, and has a positive significance for prognosis 14. Additionally, the expression of the KCNK5 gene can be increased by the activation of the estrogen α receptor, and silencing its expression can inhibit the proliferative capacity of estrogen-induced breast cancer cell lines 15. In this study, we performed comprehensive bioinformatics analysis and immunohistochemical staining of KCNKs expression in thyroid cancer and evaluated whether their expression is related to clinical parameters of thyroid cancer patients. This has extremely important clinical and scientific significance for exploring the new pathogenesis and finding new potential therapeutic targets of thyroid carcinoma.

## Materials and Methods

### TCGA data and cBioPortal

The mRNA expression and clinical information data of KCNKs in 512 thyroid papillary carcinoma samples and 18 normal tissues was obtained from the Cancer Genome Atlas (TCGA) database 16. The cBioPortal dataset for papillary thyroid carcinoma (TCGA, Cell, 2014) (including data from 388 pathology reports) was then used for further analysis and visualization of the KNCK family.

### Tissue samples

The PTC tissue microarray (HThy-Pap120CS-01) consisted of 120 points in 58 cases were bought from Shanghai Outdo Biotech CO., LTD. All patients enrolled into our current study were pathologically diagnosed as PTC with no extra treatment before surgery.

### UALCAN analysis

UALCAN based on the TCGA database can help scholars analyze clinical data of 31 common cancers online, allowing us to easily obtain the expression of specific genes in different tissues, and then analyze the gene expression in different TNM stages. In addition, the website can also analyze gene survival results and methylation levels online 17. In this study, the mRNA levels of KNCKs in thyroid carcinoma samples were compared with that in the normal tissues and the expression of KNCKs in different clinical stages was analyzed. Student's t-test was performed to generate a p-value.

### The Kaplan-Meier plotter

The overall survival (OS) of KCNKs was conducted in the Kaplan-Meier plotter in patients with thyroid carcinoma which contains 502 patients from TCGA 18. The overall survival curves were analyzed based on differential expression of KNCK family genes. A log P-value <0.05 was set as statistically significant.

### GeneMANIA analysis

GeneMANIA is a flexible website that can predict the function of genes for scholars, analyze the priority of gene set functions, and then build a protein-protein interaction network 19. In addition, GeneMANIA can also provide weights for specific functions of genes and identify similar networks of genes. In this experiment, we used GeneMANIA to predict the role of KCNK family genes and genes with similar functions, and to construct a protein-protein interaction network for visualization.

### Functional enrichment

FunRich is a customizable, free software tool for functional enrichment analysis of genes and miRNAs (including Biological Process; Cellular Component; Molecular Function; Pathway) 20. We used FunRich to perform functional enrichment analysis of KCNKs with their closely related TOP-50 genes. Likewise, Cytoscape plugin-clueGO was used again to analyze the functional enrichment analysis of KNCK family genes and their closely related genes 21. GSCALite is a friendly, a cancer multi-omics database for genomics (including mRNA, SNV, CNV, Methylation, Cancer pathway activity, miRNA network, Gene regulatory network by miRNAs, Drug analysis, GTEx) analysis, based on TCGA data in 33 cancers 22. Finally, in order to clarify whether KCNKs play a role in the cancer pathway, we used the GSCALite platform to analyze the pathway vitality of KCNKs online.

### LinkedOmics Database Analysis

The correlated genes of KCNK2, KCNK4, KCNK5, KCNK15 in human thyroid carcinoma were analyzed by using LinkedOmics. The LinkedOmics database is a website for multi-omics and clinical information analysis of 32 kinds of cancers 23. The genes correlated with KCNK2, KCNK4, KCNK5, KCNK15 in human thyroid carcinoma were visualized in the LinkFinder module. The kinase-target, miRNA-target and transcription factor-target analysis of KCNK2, KCNK4, KCNK5, KCNK15 associated genes were analyzed in the LinkInterpreter module.

### TIMER analysis

The TIMER database is a website that can analyze tumor-infiltrating immune cells (TIICs) from 10,000 samples from 32 cancer types from the TCGA database 24. We analyzed the correlation of KCNK2, KCNK4, KCNK5, KCNK15 expression with the abundance of immune infiltrates by using the TIMER database.

### Immunohistochemical (IHC) staining and evaluation

Immunostaining of KCNK2, KCNK4, KCNK5, and KCNK14 was conducted using a rabbit monoclonal anti-KCNK2 antibody (1:1000, Cat. 17890-1-AP), proteintch, China), a rabbit monoclonal anti-KCNK4 antibody (1:500, Cat. 27113-1-AP), proteintch, China), a rabbit monoclonal anti-KCNK5 antibody (1:100, Cat. 14136-1-AP), proteintch, China), a rabbit monoclonal anti-KCNK15 antibody (1:1000, Cat. 18059-1-AP), proteintch, China), respectively. The overall IHC score of grading from 1 to 5 was evaluated according to the semi-quantitative immunoreactive score (IRS) scale of Remmele 25.

### Statistical methods

ROC curves were conducted using the GraphPad Prism 7 software.

## Results

### Relationship between the mRNA levels of KCNKs and the clinicopathological parameters of patients with thyroid carcinoma

**Figure 1:**
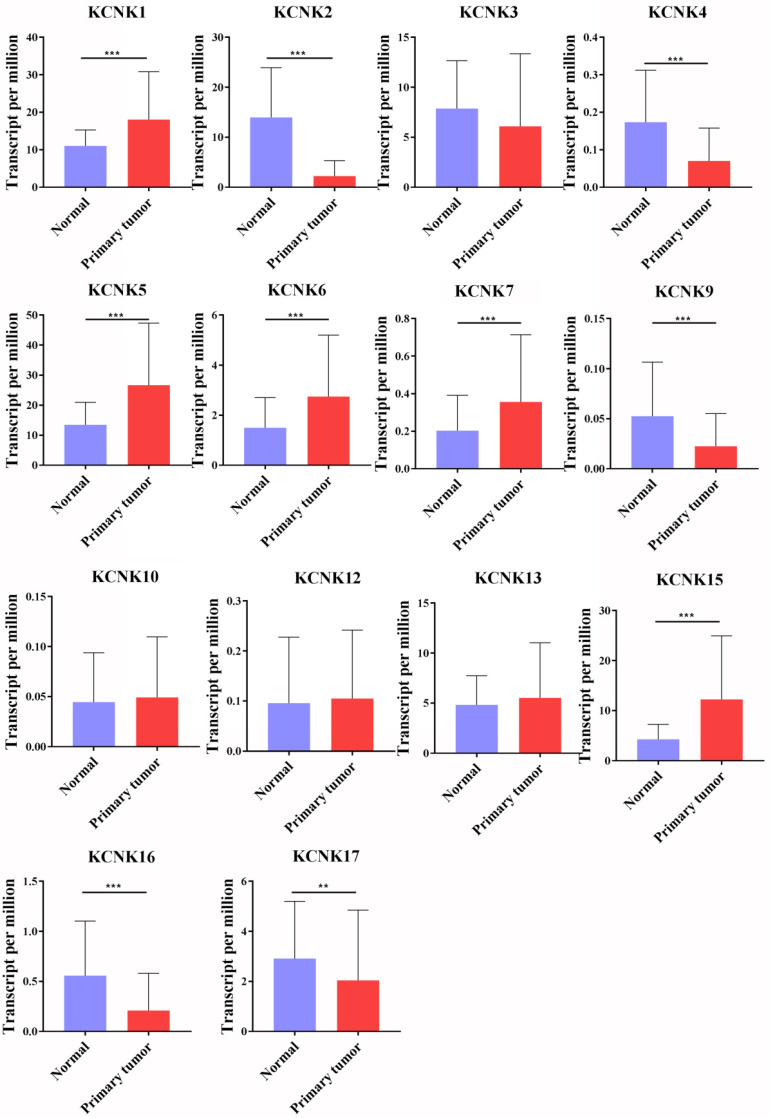
The transcription levels of KCNK factors in thyroid cancer (UALCAN). Compared with normal samples, KCNK1/5/6/7/15 mRNA was overexpressed in TC, and KCNK2/4/9/16/17 mRNA was underexpressed. **p<0.01, *** p<0.001.

So far, the KCNK family includes a total of 15 members in mammalian cells, namely KCNK1, KCNK2, KCNK3, KCNK4, KCNK5, KCNK6, KCNK7, KCNK9, KCNK10, KCNK12, KCNK13, KCNK15, KCNK16, KCNK17 and KCNK18. Using the GEPIA database and the UALCAN database, we analyzed the transcription levels of KCNKs in thyroid cancer with those in matched normal samples. Data in the GEPIA database and the UALCAN database revealed that mRNA expression of KCNK1 (p<1E-12), KCNK5 (p = <1E-12), KCNK6 (p = 2.09930000050207E-08), KCNK7 (p = 3.44069217561582E-12), and KCNK15 (p<1E-12) were significantly higher in thyroid cancer tissues than that in normal tissues, while KCNK2 (p = 1.62614366416847E-12), KCNK4 (p = 4.35290000000421E-06), KCNK9 (p = 2.136300E-03), KCNK16 (p = 1.219450E-04) and KCNK17 (p = 1.829970E-02) mRNA levels were decreased compared to normal tissues (Figure 1).

**Figure 2:**
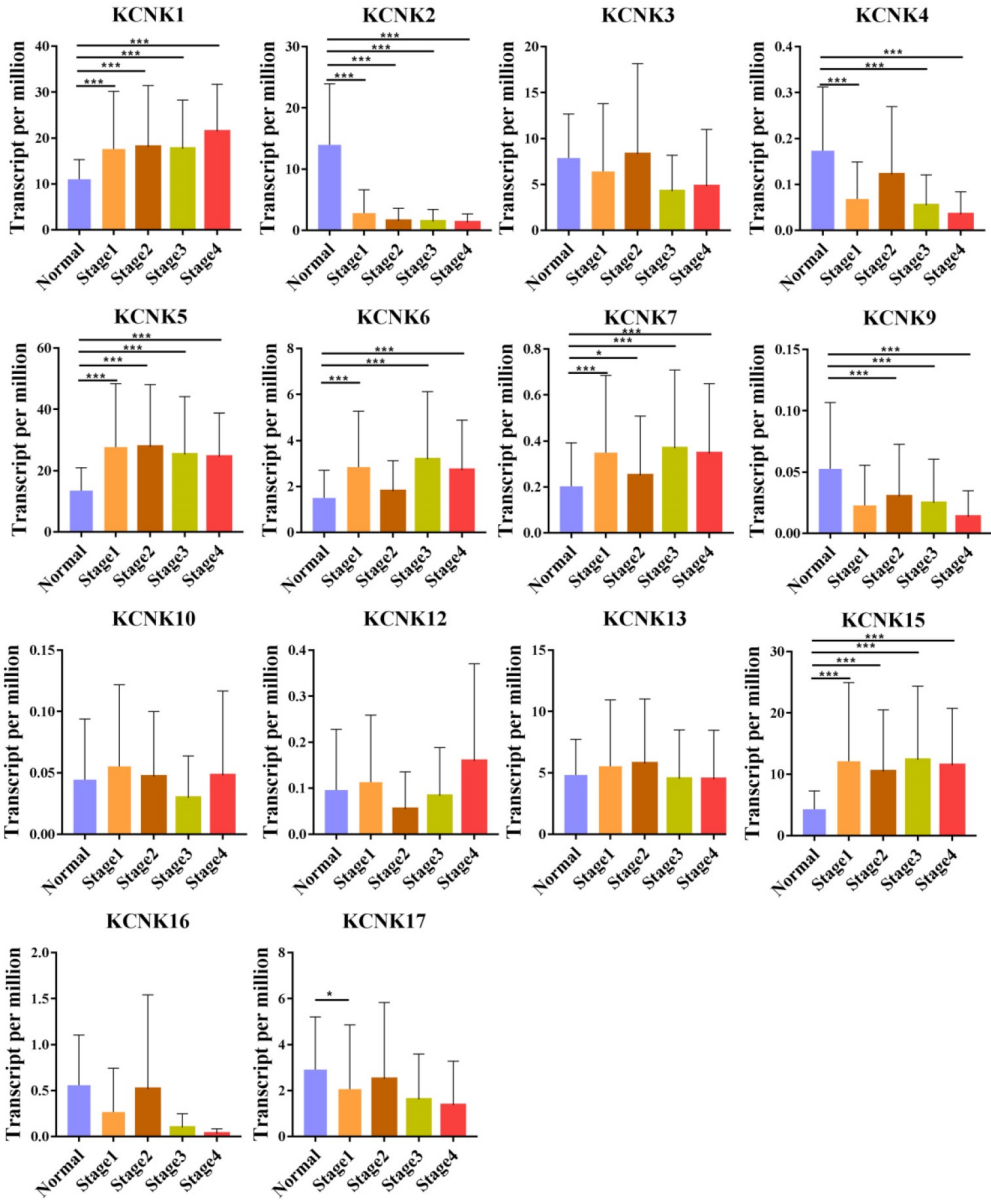
Correlation between KCNKs expression and tumor stage in TC patients (UALCAN). Compared with normal tissues, KCNK 1/ 5/6/7/15 expression was positively correlated with the clinical stage, and KCNK 2/4/9 expression was negatively correlated with the clinical stage.*p<0.05, **p<0.01, ***p<0.001.

We also analyzed the expression of KCNKs with the tumor stage for thyroid carcinoma. KCNK1, KCNK2, KCNK4, KCNK5, KCNK6, KCNK7, and KCNK15 groups significantly varied, whereas KCNK3, KCNK10, KCNK12, KCNK13 and KCNK17 did not significantly differ (Figure 2).

### KCNK2/3/4/5/12/15 levels are associated with thyroid carcinoma patient overall survival

**Figure 3:**
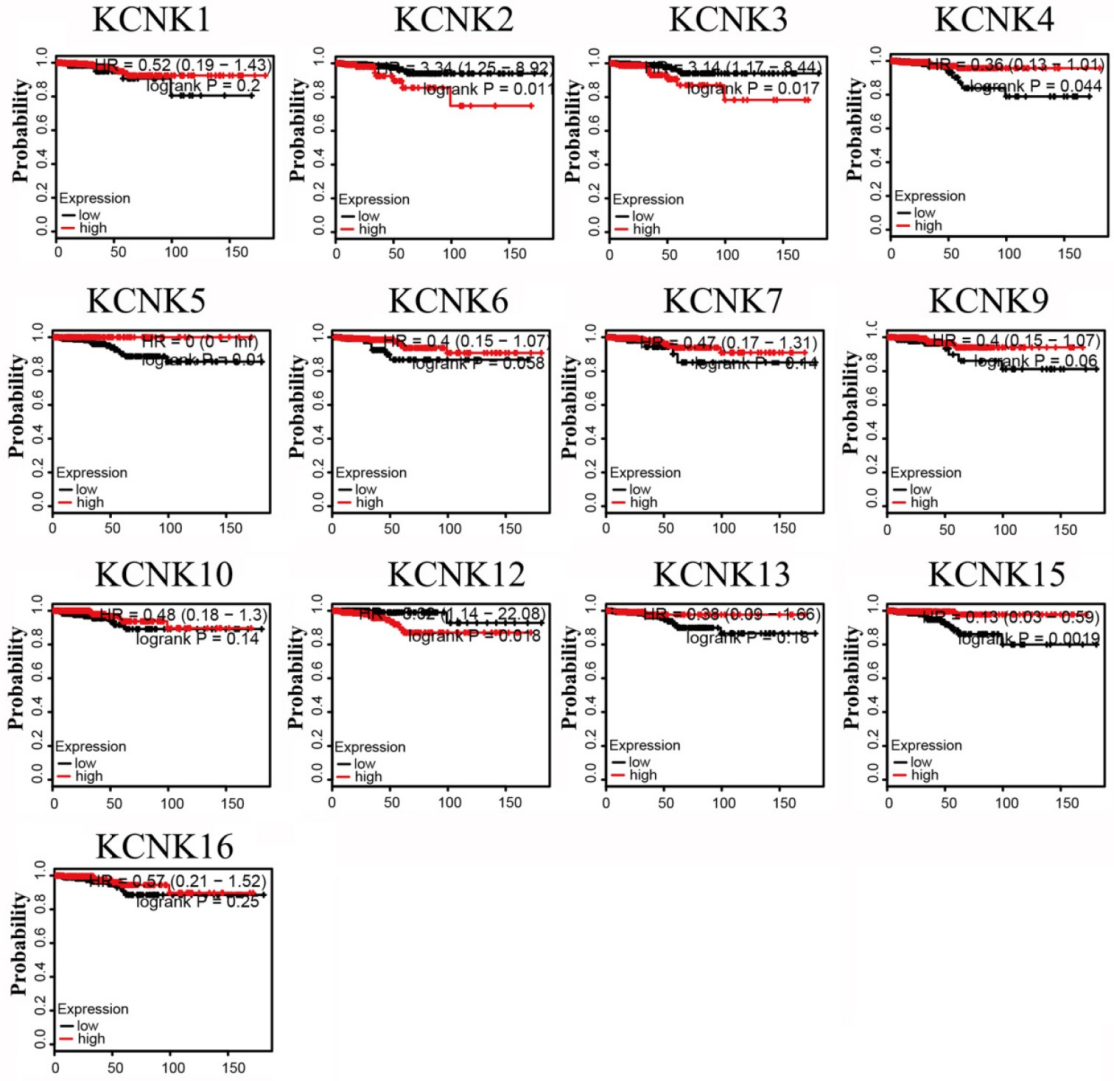
Prognostic value of mRNA expression of distinct KCNKs family members in thyroid cancer patients (Kaplan-Meier Plotter).

The prognostic values of the 10 accessible KCNKs mRNA expression levels were examined using the Kaplan-Meier Plotter in thyroid carcinoma. The results revealed that high expression of KCNK2 (HR=3.34, 95% CI: 1.25-8.92, and p=0.011), KCNK3 (HR=3.14, 95% CI: 1.17-8.44, and p=0.017), KCNK12 (HR=5.02, 95% CI: 1.14-22.08, and p=0.018) was associated with a worse overall survival rate in the thyroid carcinoma patients while high expression of KCNK4 (HR=0.36, 95% CI: 0.13-1.01, and p=0.044), KCNK5(HR=0, 95% CI: 0-Inf, and p=0.01), and KCNK15(HR=0.13, 95% CI: 0.03-0.59, and p=0.0019) was associated with a longer overall survival rate (Figure 3). However, KCNK1, KCNK6, KCNK7, KCNK9, KCNK10, KCNK13 and KCNK16 mRNA expressions were not related to the prognosis of patients with thyroid cancer. Interestingly, the results that the low mRNA levels of KCNK2 and KCNK3 were related to longer overall survival in thyroid cancer patients was inconsistent with different analyses of the UALCAN database, which showed low KCNK2 and KCNK3 expression in thyroid cancer. Besides, we found that KCNK5 and KCNK15, which were highly expressed in thyroid cancer, had a better overall survival rate.

### Predicted functions and pathways of KCNKs and their frequently altered neighbor genes with thyroid carcinoma

**Figure 4:**
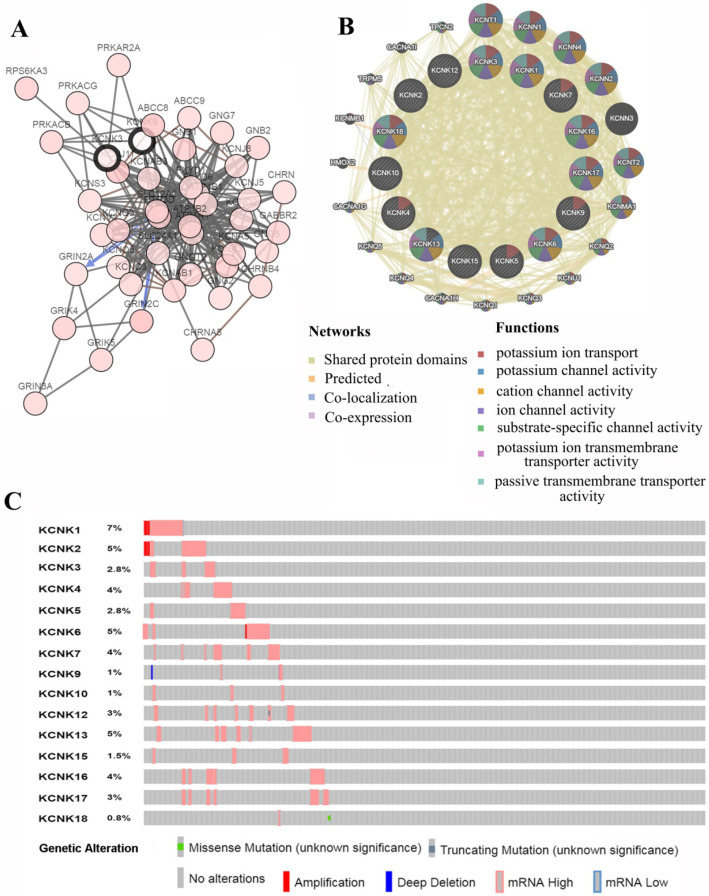
Genetic mutations in KCNKs and their association neighbor genes network of PTC patients (cBioPortal and GeneMANIA). (**A**) The network contains 65 nodes, including KCNKs factors and the 50 most frequently altered neighbor genes. (**B**) Protein-protein interaction network of KCNKs. (**C**) Summary of alterations in differently expressed KCNKs in PTC.

We analyzed the KCNKs alterations, networks by using the cBioPortal and GeneMANIA online tool for papillary thyroid carcinoma (TCGA, Cell, 2014). KCNKs were altered in 129 samples (33%) of 388 patients with papillary thyroid carcinoma (33%). Provisional datasets of TCGA were used to analyze the genetic alterations of KCNKs. As a result, KCNK1, KCNK2, KCNK3, KCNK4, KCNK5, KCNK6, KCNK7, KCNK9, KCNK10, KCNK12, KCNK13, KCNK15, KCNK16, KCNK17 and KCNK18 were altered in 5%, 2.8%, 4%, 2.8%, 5%, 4%, 1%, 1%, 3%, 5%, 1.5%, 4%, 3% and 0.8% of the queried papillary thyroid carcinoma samples, respectively (Figure 4C). To shed light on the underlying mechanism of KCNKs expression in thyroid carcinoma, a gene regulation network containing KCNKs, as shown in Figure 4A and 4B, and the 50 most frequently altered neighboring genes was constructed. The results revealed that KCNKs were closely associated with potassium calcium-activated channels (KCNN1, KCNN2, KCNN3 and KCNN4), potassium voltage-gated channel family Q members (KCNQ1, KCNQ2, KCNQ3, KCNQ4 and KCNQ5) and protein kinase CAMP-activated catalytic units (PRKACA, PRKACB and PRKACG).

### Functional enrichment analysis

**Figure 5:**
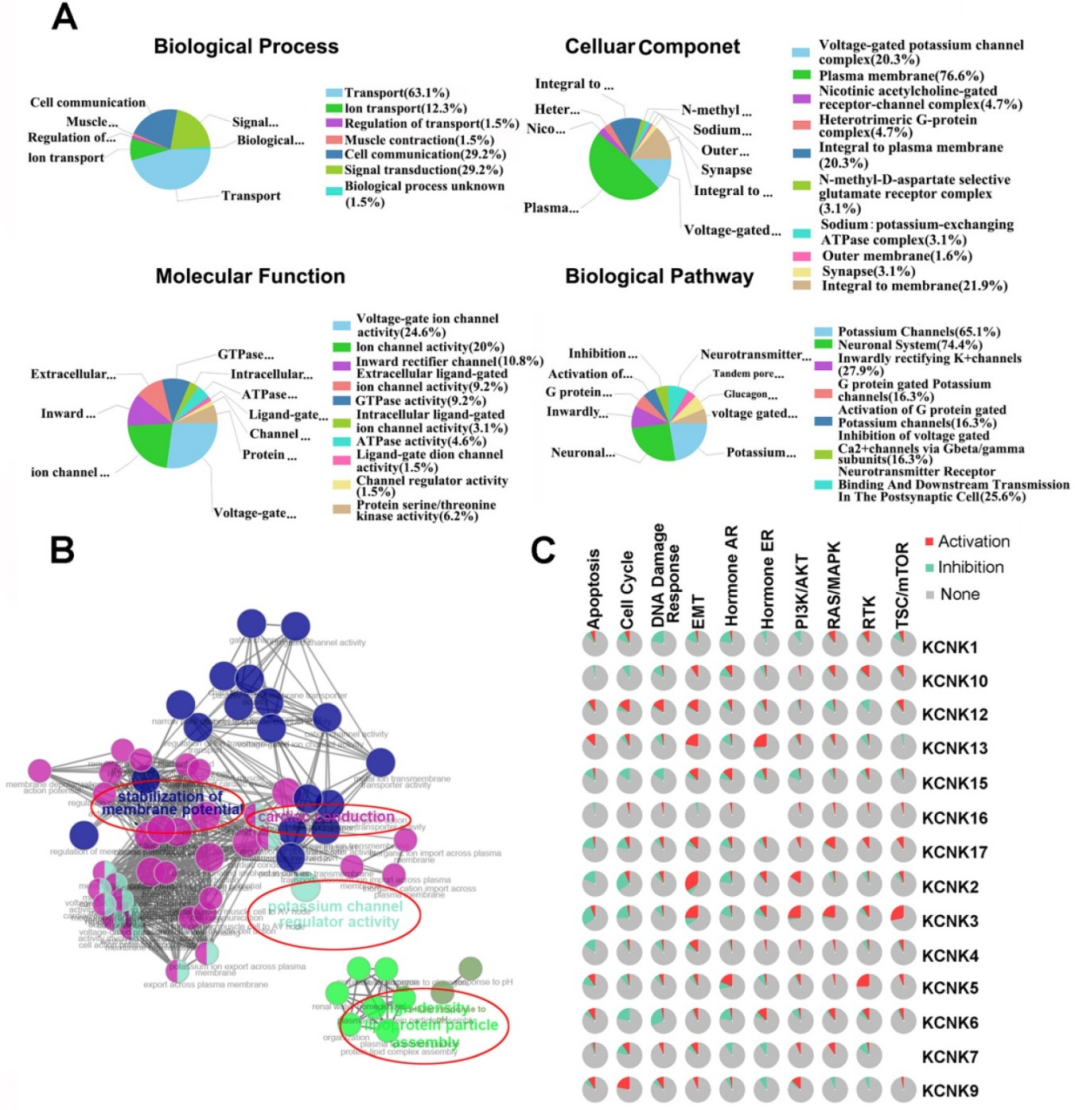
The function of KCNKs factors and the significant genes associated with KCNKs changes were analyzed by various analysis tools. (**A**) Funrich analysis. (**B**) Cluego analysis. (**C**) GSCALite pathway analysis.

The functions of KNCKs and the genes significantly related to KNCKs alterations were predicted by Funrich software. Funrich software predicted the function of these genes on the basis of four aspects, including biological processes, cellular components, molecular functions and biological pathways. Among the 5 most highly enriched functions in the BP category, transports (63.1%), cell communication (29.2%), signal transduction (29.2%), ion transport (12.3%), and regulation of transport (1.5%) were associated with the tumorigenesis of thyroid carcinoma. The plasma membrane (76.6%), integral to membrane (21.9%), voltage-gated potassium channel complex (20.3%), integral to plasma membrane (20.3%), heterotrimeric G-protein complex (4.7) were the five most highly involved items in the CC category. In the molecular function, the roles of these genes are enriched in voltage-gated ion channel activity (24.6%), ion channel activitity (20%), inward rectifier channel (10.8%), extracellular ligand-gated ion channel activity (9.2%) and GTPase activity (9.2%). Seven biological pathways related to the functions of KCNKs alterations in thyroid carcinoma were investigated through KEGG analysis (Figure 5A). Figure 5B showed the results of a feature-rich analysis obtained from Clue Go. As shown in Figures 5A and 5B, the functions of KCNKs and its neighboring genes are mainly concentrated on regulation of potassium channel regulator activity and stabilization of membrane potential. In order to further determine whether KCNK family genes are involved in cancer-related pathways and cancer processes, we performed KCNK genes enrichment analysis and the results were visualized through the GSCALite platform. As shown in Figure 5C, all KCNK genes were involved in the regulation of cancer-related pathways and cancer processes, specifically in the activation or suppression of these pathways and processes.

In summary, by analyzing the differential expression of KCNKs genes, clinical staging analysis, prognosis analysis and functional enrichment analysis, we found that only four genes, KCNK2, KCNK4, KCNK5, and KCNK15, may play a role in the carcinogenesis of thyroid cancer. Next, we explored the possible mechanisms of these four genes.

### Immune cell infiltration of KCNK2/4/5/15 in patients with thyroid carcinoma

**Figure 6:**
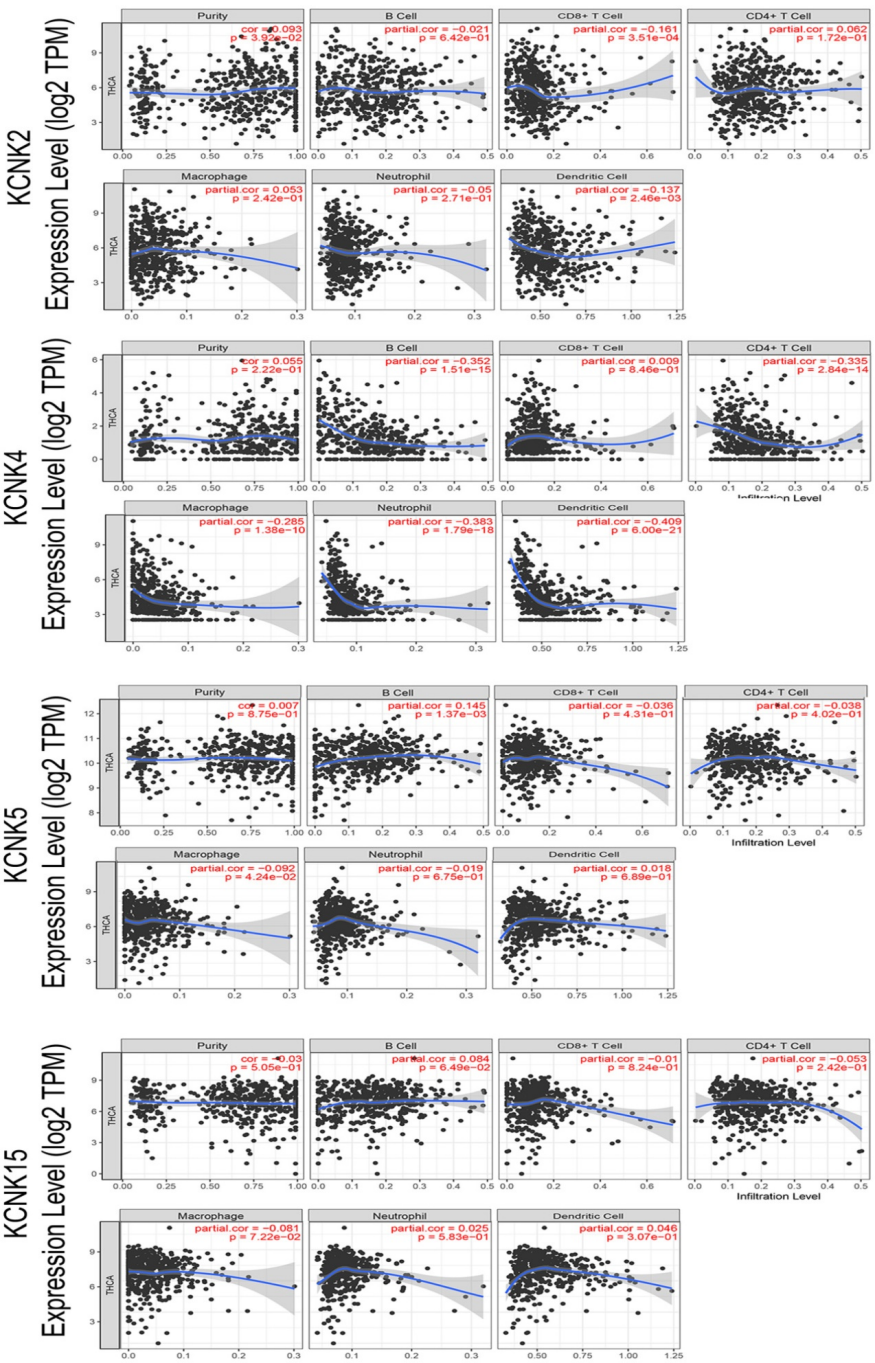
Correlations between expression of KCNK2/4/5/15 with immune infiltration level in TC. KCNK2 expression was negatively associated with the infiltration of CD8+ T cells and dendritic cell, and positively associated with the infiltration of purity. The level of KCNK4 expression negatively correlated with the infiltration levels of B cells, CD4+ T cells, macrophages, neutrophils, and dendritic cell in thyroid carcinoma tissues. KCNK5 expression was negatively associated with the infiltration of macrophage, and positively associated with the infiltration of B cells.

With the comprehensive and thorough study of tumor immune cell infiltration, immune escape is currently considered to be one of the important mechanisms of tumorigenesis and development 26. Then, we used the TIMER database to explore the relationship between KCNK2 /4/5/15 expression and infiltrating immune cells in thyroid cancer (Figure 6). KCNK2 expression was negatively associated with the infiltration of CD8+ T cells (Cor = -0.161, p = 3.51e-04) and dendritic cell (Cor = -0.137, p = 2.46e-03), and positively associated with the infiltration of purity (Cor = 0.093, p = 3.92e-02). The level of KCNK4 expression negatively correlated with the infiltration levels of B cells (Cor =-0.352, p=1.51e-15), CD4+ T cells (Cor =-0.335, p=2.84e-14), macrophages (Cor =-0.285, p=1.38e-10), neutrophils (Cor =-0.383, p=1.79e-18), and dendritic cell (Cor = -0.409, p = 6e-21) in thyroid carcinoma tissues. KCNK5 expression was negatively associated with the infiltration of macrophage (Cor = -0.092, p = 4.24e-02), and positively associated with the infiltration of B cells (Cor = 0.145, p = 1.37e-03). However, data from the TIMER data indicated that KCNK15 was not associated with immune cell infiltration.

### Regulators and validation of KCNK2/4/5/15 in patients with thyroid carcinoma

**Figure 7:**
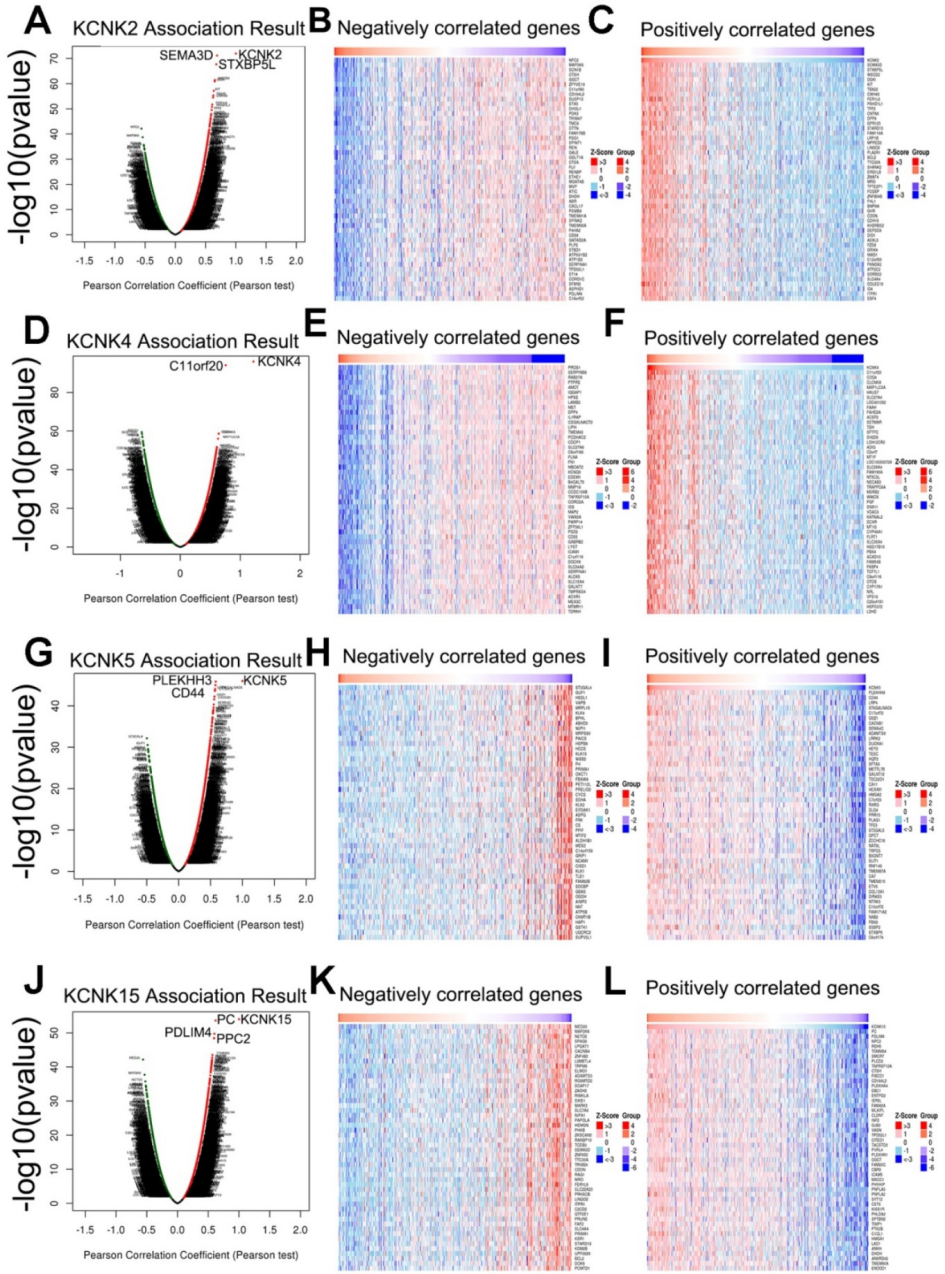
Genes differentially expressed in correlation with KCNK2/4/5/15 in thyroid carcinoma (LinkedOmics). (**A, D, G, J**) The correlation between KCNK2/4/5/15 and differentially expressed genes was analyzed by the Pearson test analysis in volcano plots. (**B, C, E, F, H, I, K, L**) The heatmap showing the top 50 genes negatively or positively correlated with KCNK2/4/5/15.

Next, the genes related to KCNK2/4/5/15 and differentially expressed in thyroid cancer were collected by LinkedOmics to investigate the mechanism of KCNK2/4/5/15 in thyroid cancer. As shown in Figure (7A, 7D, 7G, 7J), genes significantly related to KCNK2/4/5/15 were shown in the volcano plots, where the red dots represented positive correlation genes and the green dots represented negative correlations genes. And the top-50 genes that were positively and negatively correlated with KCNK2 /4/5/15 were shown in Figure 7B, 7C 7E, 7F, 7H,7I, 7H, 7L, respectively.

**Figure 8:**
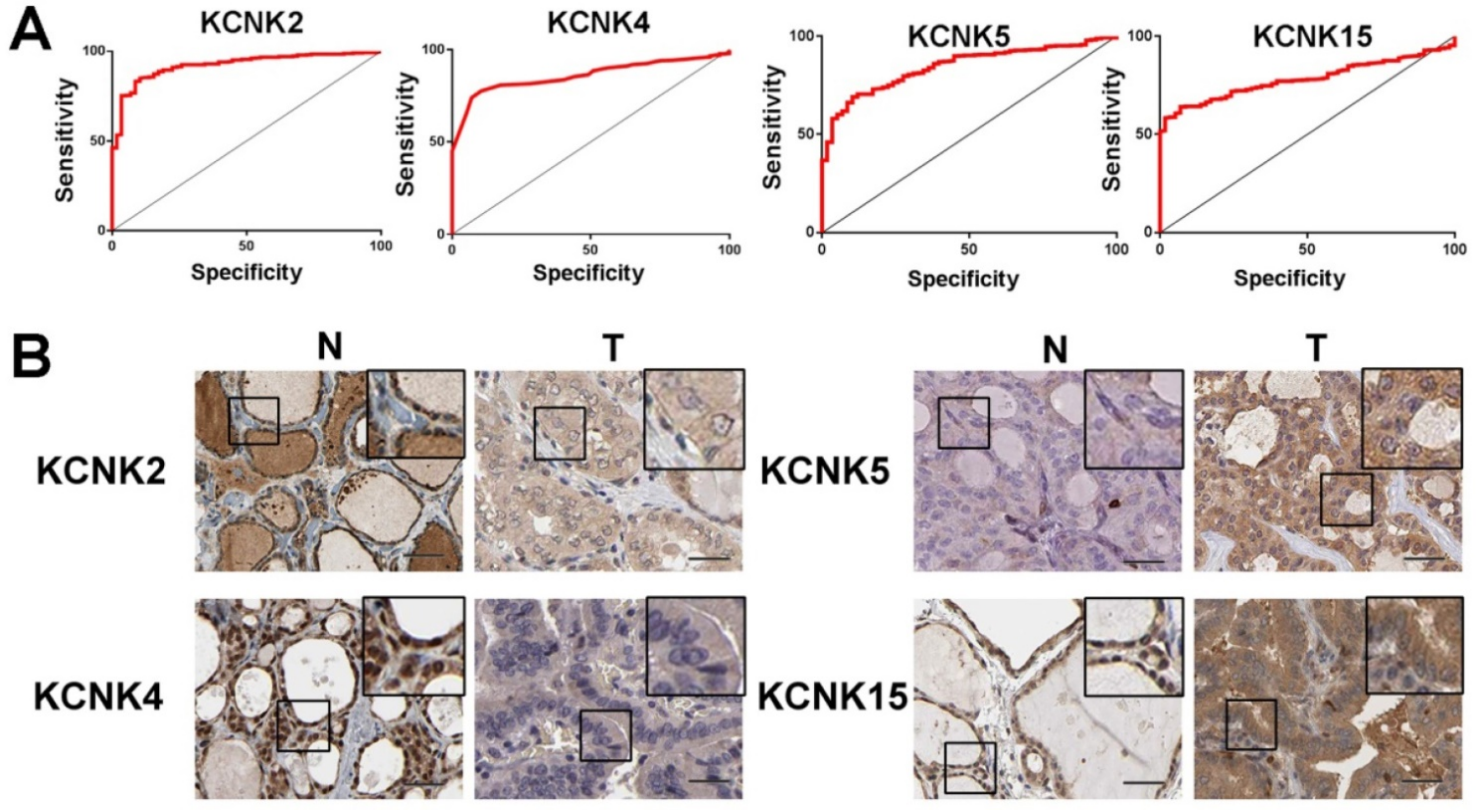
ROC curves and immunohistochemistry results of KCNK2, KCNK4, KCNK5, KCNK15. (**A**) KCNK2, AUC=0.9229 (95%CI: 0.8933-0.9524), p < 0.0001; KCNK4, AUC=0.8591 (95% CI: 0.8246-0.8935), p <0.0001; KCNK5, AUC=0.8445 (95% CI: 0.8038-0.8852), p <0.0001; KCNK15, AUC=0.7815 (95% CI: 0.742-0.821) , p <0.0001. (**B**) KCNK5 and KCNK15 proteins were higher in PTC tissues and KCNK2 and KCNK4 proteins were lower in PTC, compared to tumor-adjacent tissues, Bar=20 µm.

To further discover the targets of KCNK2/4/5/15 in thyroid carcinoma, we analyzed the possible kinase, miRNA and transcription factor target using LinkedOmics database. As summarized in Table 1, for KCNK2, the most correlated kinase-targets was PDGFRB; the most correlated microRNA-targets were MIR-221, MIR-222, MIR-144, MIR-500, MIR-34B; the most correlated transcript factor-targets were CAGNYGKNAAA_UNKNOWN, YGTCCTTGR_UNKNOWN and AAAYWAACM_V$HFH4_01. For KCNK4 (Table 2), the most correlated kinase-targets network were MAPKAPK2, ABL1, TBK1, PDGFRB and JAK2; the most correlated microRNA-targets were MIR-26A, MIR-26B, MIR-519C/MIR-519B/MIR-519A, MIR-186, MIR-448, MIR-200B, MIR-200C, MIR-429, MIR-203, MIR-330, MIR-17-5P, MIR-20A/MIR-20B, MIR-106A/MIR-106B, MIR-519D, MIR-19A/MIR-19B, MIR-22, MIR-30A-5P/MIR-30E-5P, MIR-30B/MIR-30C/MIR-30D, MIR-493; the most correlated transcript factor-targets were V$NFKB_Q6, V$EGR2_01, TTCYNRGAA_V$STAT5B_01, TGANTCA_V$AP1_C, V$IRF1_Q6, V$HLF_01, V$PEA3_Q6. For KCNK5 (Table 3), the most correlated microRNA-targets was MIR-296; the most correlated transcript factor-targets were V$EGR1_01, V$STAT_01, V$LFA1_Q6, V$NFKB_Q6, V$MYOGENIN_Q6, V$AP2ALPHA_01, V$SREBP1_Q6, V$FAC1_01. For KCNK15 (Table 4), the most correlated microRNA-targets were MIR-221, MIR-222, MIR-34B; the most correlated transcript factor-targets were V$BACH2_01, TGANTCA_V$AP1_C, GGGNNTTTCC_V$NFKB_Q6_01, V$AP1_Q4_01, V$BACH1_01, V$AP1_C, V$PPAR_DR1_Q2, V$AP1_Q6, V$AP1FJ_Q2, V$TEF1_Q6, V$AP1_Q2_01, V$AP1_01, V$SREBP1_Q6, V$AP1_Q4, CCANNAGRKGGC_UNKNOWN, ACCTGTTG_UNKNOWN, TGASTMAGC_V$NFE2_01. Additionally, ROC curves were conducted to evaluate the diagnostic effect of KCNK2/4/5/15 in thyroid carcinoma. As shown in Figure 8A and table 5, KCNK 2/4/5/15 can effectively distinguish thyroid carcinoma patients. The AUC of KCNK2 was 0.9229 (95% CI: 0.8933-0.9524, p <0.0001); The AUC of KCNK4 was 0.8591 (95% CI: 0.8246-0.8935, p <0.0001); The AUC of KCNK5 was 0.8445 (95% CI: 0.8038-0.8852, p <0.0001); AUC of KCNK15 was 0.7815 (95% CI: 0.742-0.821, p <0.0001). Finally, we assessed the expression of KCNK2/4/5/15 in PTC and matched tumor-adjacent tissues by immunohistochemical staining of the PTC tissue microarray with 58 patient cases. Compared with tumor-adjacent tissues, KCNK5 and KCNK15 proteins were higher in PTC tissues and KCNK2 and KCNK4 proteins were lower in PTC, especially in PTC cytoplasm (Figure 8B). Therefore, the expression of KCNK2/4/5/15 can be used as a potential diagnostic indicator in PTC.

## Discussion

According to statistics from the US Cancer Data in 2019, the number of new patients with thyroid cancer is 52,070, including 14,260 male patients, 37,810 female patients, and a total of 2,170 fatalities, indicating that the incidence of thyroid cancer is increasing rapidly 27. Because of its good prognosis, slow progress and high 10-year survival, so it has been considered as an inert tumor. However, distant metastasis occurs in 10-15% of PTC patients during the disease process, which greatly reduces the quality of life of patients, greatly increases the financial burden and affects the prognosis, and the 10-year disease-specific mortality of these patients can drop to 70% 28. Studies have shown that even in patients with Papillary Thyroid Microcarcinoma (PTMC), distant metastases can occur in 0.1% of patients, and 33% of patients will die after an average of 7.7 years of follow-up 29. It can be seen that although PTC is an inert tumor, the appearance of distant metastatic lesions will be an important turning point in the survival and prognosis of PTC patients. Thyroid cancer can now be treated with surgical resection, radioactive iodine therapy, and drug targeting, but it usually cannot be cured. Therefore, it is particularly important to investigate the molecular mechanism of PTC lymphatic metastasis and distant metastasis from a genetic perspective.

Tumor microenvironment is composed of many components, in which immune cells are the main components, and they mediate the generation and development of tumor through regulating tumor microenvironment. There is increasing evidence that potassium channels (including KCNKs) can activate and modulate the electrophysiological activity of immune cells and thus contribute to tumorigenesis and metastasis by influencing the tumor microenvironment. 30. Some studies have reported that KCNKs play important roles in cellular behaviors related to tumor progression, including regulating cell proliferation, migration, apoptosis, and angiogenesis. For example, Katharina et al showed that silencing KCNK3 in A549 cells can reduce the proliferation and enhance the apoptosis of lung cancer cells 31. And it is reported that KCNK9 plays a key role in promoting the survival and growth of breast cancer cells, and monoclonal antibody-based KCNK9 targeting has therapeutic prospects in the treatment of primary tumors and metastases, by inhibiting KCNK9 channel function or activating anti-tumor immune response 32. However, the diagnostic value, biological function, and prognostic value of KCNKs have not been well characterized in PTC.

In this study, we first investigated the relationship between the expression of KCNKS and the pathological staging of thyroid carcinoma. We found that 10 KCNK family factors were abnormally expressed compared to normal thyroid tissue (upregulation of KCNK1, KCNK5, KCNK6, KCNK7 and KCNK15; downregulation of KCNK2, KCNK4, KCNK9, KCNK16 and KCNK17). Moreover, a total of 7 KCNKs were found to be closely related to the tumor stage, which were KCNK1, KCNK2, KCNK5, KCNK6, KCNK7, KCNK19, and KCNK15. 6 KCNKs were associated with the overall survival of thyroid cancer, namely KCNK2, KCNK3, KCNK4, KCNK5, KCNK12, and KCNK15. In order to understand the role of the KCNK family in PTC development, the genomic changes of KCNKs and the regulatory networks of genes closely related to KCNKs were further analyzed by cBioPortal and GeneMANIA. Next, we performed functional enrichment analysis of these genes through FunRich software and the Cytoscape plugin clueGO. Our results showed that the function of these genes were concentrated on potassium ion transport, potassium channel activity, cation channel activity, ion channel activity, substrate-specific channel activity, potassium ion transmembrane transporter activity and passive transmembrane transporter activity. We noted that most of these effects were focused on ion channels or ionic activity, and it had been suggested that K+ ion channels, which were important ion channels in lymphocyte membranes, were participating in the regulation of immune microenvironment in cancers 33, 34. Besides, based on the analysis of cancer-related processes and pathways, KCNK family genes were involved in the activation or suppression of cell cycle, DNA damage response, epithelial-mesenchymal transformation, hormone AR/ER, PI3K, MAPK, RTK and mTOR signaling pathways. These data, taken together, suggested that KCNK2/4/5/15 may play a regulatory role in the carcinogenesis and metastasis of PTC and may be a potential therapeutic target.

To further understand the role of KCNK 2/4/5/15 in regulating PTC carcinogenesis, we first analyzed the co-expressed genes of these four KNCK genes using the function module of LinkedOmics. The results indicate that KCNK2, KCNK4, KCNK5, KCNK15 have extensive effects on the transcriptome. Next, the transcription factors, kinases and miRNAs of these four genes were analyzed to explore their specific regulatory role in PTC. In the transcription factor network, we found that HFH4 may be a key transcription factor that regulated KCNK2; For KCNK4, AP1, IRF1, NFκB and STAT may be key transcription factors; STAT, NFKB, and EGR1 may be key transcription factors regulating KCNK5; And AP1 may be a key transcription factor for KCNK15. FOXJ1, also known as HNF-4, is thought to inhibit tumor growth by resisting NF-κB activity, and FOXJ1 is ubiquitous in hypermethylated tumors and it is suggested that FOXJ1 silencing is an important potential factor in breast tumor formation 35. Researchers have pointed out that activated AP1 can regulate the expression of many genes, participate in transformation, proliferation, differentiation, apoptosis and other biological functions, especially activate many tumor-related genes, and promote the occurrence and evolution of tumors 36, 37. Yang et al reported that STAT3 is activated for only a few minutes to several hours under physiological conditions, but in malignant tumors, STAT3 is continuously activated and JAK2/STAT3 signaling pathway can interact with many cytokines in the process of tumor growth, invasion, metastasis and apoptosis 38. In this study, we predicted both kinase JAK2 and transcription factor STAT for KCNK4 in PTC, which may indicate that these two factors may play a role in the regulation of PTC. Activation of nuclear transcription factor (NFkB) can promote the transcription of apoptotic genes, leading to the proliferation of tumor cells, and affect the sensitivity of tumor cells to chemotherapy and the prognosis of patients 39-41. EGR1 is a transcription factor with three zinc finger structures, which can compete with SP1 or synergistically regulate the transcription of target genes. In terms of its mechanism, Sp1 competes with EGR-1 for binding to the ADA gene promoter, Sp1 stimulates transcription through the ADA promoter, and EGR-1 inhibits transcription by replacing the binding of Sp1 42. In summary, combining our experimental results and considering that multiple transcription factors may interact with each other, we considered AP1, NFκB, STAT, and EGR1 as key transcription factors for KCNK 2/4/5/15 in PTC. In addition, considering that the KCNK family may have a role in regulating the infiltration of cancer immune cells, we tested whether these four genes are involved in immune regulation. Finally, the expression of KCNK2/4/5/15 was detected by immunochemical staining of PTC tissue chips and was consistent with the results obtained by our bioinformatics analysis. Therefore, these four KCNK factors can be used as potential diagnostic markers and drug targets for PTC.

At the same time, there are still some limitations in our experiments. First, the data downloaded from the TCGA database may contain very few cases of thyroid adenoma genes. Second, the KCNK 2/4/5/15 genes do not regulate all the tumor-inhibiting immune cells. Third, our analysis of the transcription factors in PTC still needs to be tested experimentally to verify our hypothesis.

## Figures and Tables

**Table 1:** The Kinase, miRNA and transcription factor-target networks of KCNK2 in Thyroid carcinoma (LinkedOmics)

Enriched Category	Geneset	LeadingEdgeNum	FDR
Kinase Target	Kinase_PDGFRB	15	0.028289
miRNA Target	ATGTAGC, MIR-221, MIR-222	49	0.016865
	ATACTGT, MIR-144	59	0.021684
	AGGTGCA, MIR-500	42	0.044172
	ACTGCCT, MIR-34B	57	0.047843
Transcription Factor Target	CAGNYGKNAAA_UNKNOWN	24	0.046862
	YGTCCTTGR_UNKNOWN	19	0.048575
	AAAYWAACM_V$HFH4_01	55	0.049031

**Table 2:** The Kinase, miRNA and transcription factor-target networks of KCNK4 in Thyroid carcinoma (LinkedOmics)

Enriched Category	Geneset	LeadingEdgeNum	FDR
Kinase Target	Kinase_MAPKAPK2	12	0.012078
	Kinase_ABL1	83	0.012078
	Kinase_TBK1	15	0.013084
	Kinase_PDGFRB	15	0.016305
	Kinase_JAK2	23	0.017361
miRNA Target	TACTTGA,MIR-26A,MIR-26B	285	0
	TGCACTT,MIR-519C,MIR-519B,MIR-519A	145	0.0040747
	ATTCTTT,MIR-186	252	0.0054330
	ATATGCA,MIR-448	200	0.0065195
	CAGTATT,MIR-200B,MIR-200C,MIR-429	442	0.010532
	CATTTCA,MIR-203	98	0.010757
	TGCTTTG,MIR-330	108	0.011002
	GCACTTT,MIR-17-5P,MIR-20A,MIR-106A,MIR-106B,MIR-20B,MIR-519D	185	0.011409
	TTTGCAC,MIR-19A,MIR-19B	151	0.011557
	GGCAGCT,MIR-22	72	0.012061
	TGTTTAC,MIR-30A-5P,MIR-30C,MIR-30D,MIR-30B,MIR-30E-5P	207	0.012278
	ATGTACA,MIR-493	89	0.012315
Transcription Factor Target	V$NFKB_Q6	85	0.021987
	V$EGR2_01	53	0.022903
	TTCYNRGAA_V$STAT5B_01	91	0.03195
	TGANTCA_V$AP1_C	298	0.044723
	V$IRF1_Q6	81	0.04571
	V$HLF_01	70	0.045806
	V$PEA3_Q6	76	0.049471

**Table 3:** The Kinase, miRNA and transcription factor-target networks of KCNK5 in Thyroid carcinoma (LinkedOmics)

Enriched Category	Geneset	LeadingEdgeNum	FDR
miRNA Target	GGGGCCC, MIR-296	24	0.033591
Transcription Factor Target	V$EGR1_01	75	0.0061627
	V$STAT_01	74	0.0063144
	V$LFA1_Q6	74	0.0063388
	V$NFKB_Q6	71	0.0070995
	V$MYOGENIN_Q6	70	0.0077067
	V$AP2ALPHA_01	76	0.0081348
	V$SREBP1_Q6	67	0.0083197
	V$FAC1_01	57	0.010987

**Table 4:** The Kinase, miRNA and transcription factor-target networks of KCNK15 in Thyroid carcinoma (LinkedOmics)

Enriched Category	Geneset	LeadingEdgeNum	FDR
miRNA Target	ATGTAGC, MIR-221, MIR-222	50	0
	ACTGCCT, MIR-34B	71	0.041457
Transcription Factor Target	V$BACH2_01	59	0.01702
	TGANTCA_V$AP1_C	251	0.01722
	GGGNNTTTCC_V$NFKB_Q6_01	47	0.018416
	V$AP1_Q4_01	72	0.018416
	V$BACH1_01	71	0.02288
	V$AP1_C	55	0.023438
	V$PPAR_DR1_Q2	59	0.030762
	V$AP1_Q6	67	0.030916
	V$AP1FJ_Q2	64	0.032739
	V$TEF1_Q6	45	0.033124
	V$AP1_Q2_01	70	0.034396
	V$AP1_01	61	0.034425
	V$SREBP1_Q6	73	0.035157
	V$AP1_Q4	66	0.035827
	CCANNAGRKGGC_UNKNOWN	29	0.037529
	ACCTGTTG_UNKNOWN	45	0.038505
	TGASTMAGC_V$NFE2_01	55	0.043035

**Table 5:** The ROC test results of KCNKs

Gene	Area	*P* value	95% Confidence Interval	
			Lower Bound	Upper Bound
KCNK2	0.9229	<0.0001	0.8933	0.9524
KCNK 4	0.8591	<0.0001	0.8246	0.8935
KCNK 5	0.8445	<0.0001	0.8038	0.8852
KCNK15	0.7815	<0.0001	0.742	0.821
